# A Knowledge-Based Assessment of Dermatological Care for Transgender Women

**DOI:** 10.1089/trgh.2018.0001

**Published:** 2018-05-01

**Authors:** James J. Sabra, Celestia X. Fang, Roopal V. Kundu

**Affiliations:** Department of Dermatology, Northwestern University Feinberg School of Medicine, Chicago, Illinois.

**Keywords:** dermatology, hair removal, hormone therapy, transgender health, women's health

## Abstract

Dermatologic care plays an important role in the transitioning process for transgender women, with changes occurring to the skin from hormone therapy (HT) and gender affirming procedures. We sought to identify knowledge gaps in a group of transgender women pertaining to both skin and hair changes during the transitioning process. The study was conducted as a cross-sectional survey. Our results demonstrate potential gaps in knowledge that transgender women have regarding HT and gender affirming procedures.

## Introduction

The use of hormone therapy (HT) (e.g., estrogen, testosterone blockers, and progesterone) in transgender women can lead to various cutaneous changes: a decrease in male-pattern hair growth, xerosis, or brittle nails.^1,2^ Although a decrease in male-pattern hair growth may be a desired cutaneous change for transgender women on HT, studies have shown that HT alone may not decrease hair growth sufficiently.^3^ In light of this, some transgender women seek hair reduction procedures to achieve more desirable hair-loss effects. Hair reduction procedures (e.g., laser hair reduction or electrolysis hair removal) have recently been identified as the most sought-after category of dermatological procedure for transgender women.^4^ However, there is currently no data to support whether or not transgender women know either the potential skin changes of HT or the outcomes of hair removal procedures. This study seeks to identify the knowledge transgender women have on cutaneous changes that occur during HT as well as basic hair reduction procedure knowledge.

## Methods

We conducted a single-center, cross-sectional survey study. Participants were recruited from Center on Halsted, an LGBT Community center in Chicago, IL. The recruitment period took place largely in 1 day at a public educational event held at Center on Halsted for transgender people. Inclusion criteria were as follows: (i) anyone who was 18 years or older, (ii) identified as a transgender woman, (iii) and was able to complete a survey in English. The survey contained 34 multiple-choice questions, divided into three main sections: (i) demographic information and personal HT/gender-affirming procedure history, (ii) knowledge of skin changes from HT, and (iii) knowledge of gender affirming procedures (see Survey for list of questions in the Supplementary Data). Survey data were analyzed to calculate scores as the percent correct for the *Knowledge of Skin Changes from HT* and the *Knowledge of Gender Affirming Procedures* sections of the survey. Data were also analyzed to identify the most common incorrect answers in these respective sections. Factors influencing knowledge outcomes (percent correct) were analyzed using Pearson correlation coefficients (PCC). The study was reviewed and approved by the Northwestern University Institutional Review Board.

## Results

Fourteen adult, transgender women were recruited. The racial and ethnic diversity of the population was as follows: white (*n*=5), black non-Hispanic (*n*=2), Pacific Islander/Asian (*n*=2), Native American/American Indian (*n*=1), and multiracial (*n*=4). Two participants were excluded from data analysis due to lack of survey completion.

The median and mean scores on the *Knowledge of Skin Changes during HT* portion of the survey were 60% and 63%, respectively (range=40−100%, standard deviation=± 18%, *n*=12). The top three gaps in knowledge for this portion were as follows: (i) skin can become drier during estrogen therapy, (ii) the greatest change in male pattern hair growth occurs on the abdomen, and (iii) lips can increase in fullness on estrogen therapy (Table 1). There was a positive correlation between participants' education levels and percent of questions answered correctly (PCC=0.56, *p*=0.060). Of note, there was no significant correlation between the length of time participants were on HT and their results on this portion of the survey (PCC=−0.039, *p*=0.001).

**Table 1.** **Top Hormone Therapy Knowledge Gaps**

Top three gaps in knowledge for skin changes during HT	Percent of participants who did not know (*n*=12)
1. Skin can become drier during estrogen therapy	58% (8/12)
2. The greatest change in male pattern hair growth occurs on the abdomen	58% (8/12)
3. Lips can increase in fullness on estrogen therapy	58% (8/12)

HT, hormone therapy.

The median and mean scores for the *Hair Removal Knowledge* portion were 40% and 47%, respectively (range=0−100%, standard deviation=± 30%, *n*=12). The top three gaps in knowledge for this portion were as follows: (i) laser hair reduction requires maintenance treatment, (ii) laser hair reduction can only reduce hair with pigment, and (iii) it is possible to perform laser hair removal on all skin tones (Table 2). There was a positive correlation between participants' education levels and the percent of questions answered correctly (PCC=0.44, *p*=0.15).

**Table 2.** **Top Hair Reduction Knowledge Gaps**

Top three gaps in knowledge for hair reduction procedures	Percent of participants who did not know (*n*=12)
1. Laser hair reduction requires maintenance treatment	83% (10/12)
2. Laser hair reduction can only reduce hair with pigment	58% (7/12)
3. It is possible to have laser hair removal performed on any skin tone	50% (6/12)

## Discussion

Although the power of this study is limited due to its size, it is important to acknowledge how the data highlight points when caring for transgender women. Our current data provide insight into transgender women's understanding into the potential physical changes that occur while on HT as well as the utilization of hair reduction procedures.

As noted above, gaps in knowledge pertaining to skin changes during HT existed in both participants we surveyed who were on HT and those who were not. As the proportion of transgender women on HT ranges in the United States from 49% to 66%, clinicians' ability to provide information on this topic could positively influence potential transition-related decisions.^5,6^ For example, knowing that HT significantly decreases hair growth on the abdomen, and that it takes ∼2 years of HT to see this decrease, could prevent transgender women from undergoing unnecessary hair removal procedures early in the transitioning process.

Furthermore, clinicians working with transgender women should understand the potential knowledge gaps for those seeking hair reduction procedures. Although gaps may vary across populations and thus differ from those highlighted by our data, discussing the strengths and limitations of available hair reduction modalities could result in higher satisfaction with procedural outcomes.

The major limitation to our study is the small sample size. Due to this limitation, it is difficult to analyze trends between participant demographic information and the knowledge we assessed. Although we did find a positive correlation between participants' education levels and the proportion of questions answered correctly, this correlation is likely due to the nature of administering a knowledge assessment to a group of women with a range of educational backgrounds.

## Supplementary Material

Supplemental data

## Abbreviations Used

HT: hormone therapy
PCC: Pearson correlation coefficients
