# Supplementary material for: A Knowledge-Based Assessment of Dermatological Care for Transgender Women
Source: Transgend Health. 2018 May 1;3(1):71–3. doi: 10.1089/trgh.2018.0001 (PMC5938641; doi:10.1089/trgh.2018.0001)
Supplement: Supplemental data [file Supp_Data.pdf]

Supplementary Data

Survey

1) What is your age?
   a) __________
2) Please specify your race/ethnicity:
   a) Black, non-Hispanic
   b) Hispanic or Latino
   c) White
   d) Pacific Islander/Asian
   e) Native American or American Indian
   f) Other (please specify) ____
3) Which of the following best match your gender identity?
   a) Man/transman
   b) Woman/transwoman
   c) Other (please specify) _____
4) Do you have health insurance?
   a) Yes
   b) No
5) If you have insurance, please specify your primary insurance:
   a) Medicare
   b) Medicaid
   c) Private Insurance
6) What is the highest level of education that you have completed?
   a) Less than high school
   b) High school or GED equivalent
   c) Some college
   d) College
   e) Graduate degree
7) Are you currently on hormone therapy (check all that apply)?
   a) Yes, estrogen therapy
   b) Yes, progesterone therapy
   c) Other (please specify) ______
   d) No, I am not on hormone therapy
8) How do you pay for your hormone therapy?
   a) Through insurance
   b) Out-of-pocket
   c) Other (please specify) _____
   d) I am not on hormone therapy
9) How long ago did you start hormone therapy?
   a) 0–3 months ago
   b) 3–6 months
   c) 6–12 months
   d) 1–2 years
   e) Greater than 2 years
   f) I am not on hormone therapy

10) Where do you obtain your hormone therapy?
    a) Primary care doctor
    b) Endocrinologist
    c) Gynecologist
    d) Online
    e) Other (please specify) ______
    f) I am not on hormone therapy
11) When you started hormone therapy, were the changes that could happen to your body explained to you in a way that you understood?
    a) Yes
    b) No
    c) I am not on hormone therapy
12) Which of the following gender affirming facial procedures have you had during your transition? (Select all that apply)
    a) Laser hair reduction
    b) Electrolysis hair removal
    c) Facial neurotoxin injections, such as Botox/Dysport/Xeomin
    d) Soft tissue facial fillers, such as Juvederm/Radiesse/Sculptra
    e) Facial reconstruction surgery
    f) Facial fat injections
    g) I have not had any facial procedures
13) Regardless of whether you have undergone any gender affirming, which one do you view as the most important to your transition?
    a) Laser hair reduction or electrolysis hair removal
    b) Facial neurotoxin injections, such as Botox/Dysport/Xeomin
    c) Soft tissue facial fillers, such as Juvederm/Radiesse/Sculptra
    d) Facial reconstruction surgery
    e) Facial fat injections
    f) Other (please specify) _______
14) Regarding your answer to question 12, why is that procedure most important to you?
    a) It is the most affordable
    b) It will give my face the appearance I desire
    c) It is the least invasive of the procedures
    d) It has the least amount of complications
    e) It will give me the fastest results
    f) Other (please specify) _______
15) Regardless of whether you have undergone any gender affirming facial procedures, which one do you view as the least important to your transition?
    a) Laser hair reduction or electrolysis hair removal
    b) Facial neurotoxin injections, such as Botox/Dysport/Xeomin
    c) Soft tissue facial fillers, such as Juvederm/Radiesse/Sculptra
    d) Facial reconstruction surgery
    e) Facial fat injections
    f) Other (please specify) _______
16) Regarding your answer to question 14, why is that procedure least important to you?
    a) It is the least affordable
    b) It will not give my face the appearance I desire
    c) It is the most invasive of the procedures
    d) It has the most amount of complications
    e) It will give me the slowest results
    f) Other (please specify) _______

The following are a set of multiple-choice questions and true or false statements regarding Hormone Replacement Therapy and facial procedures. Please answer them to the best of your ability.

17) The most common change to the skin during feminizing hormone therapy is:
    a) Drier skin
    b) More Oily skin
    c) Estrogen therapy does not change the skin
18) The most common change to the pores during feminizing hormone therapy is:
    a) Larger pores
    b) Smaller pores
    c) Estrogen therapy does not change pores
19) The most common change to the skin's sensitivity during feminizing hormone therapy is:
    a) Thicker and less sensitive skin
    b) Thinner and more sensitive skin
    c) Estrogen therapy does not change skin sensitivity
20) The most common change to male-pattern hair growth (e.g. facial, chest, and back hair) during feminizing hormone therapy is:
    a) Complete removal of male-pattern hair growth
    b) Partial reduction of male-pattern hair growth
    c) Increased appearance of male-pattern hair growth
21) The region that typically shows the greatest change in male-pattern hair growth during feminizing hormone therapy is:
    a) The face
    b) The abdomen
    c) The face and abdomen show equal changes
22) The most common change to body fat distribution during feminizing hormone therapy is:
    a) Redistribution to the abdomen
    b) Redistribution to the hips and thighs
    c) Redistribution to the arms and legs
23) The most common change to cheek fullness during feminizing hormone therapy is:
    a) Increased cheek fullness
    b) Decreased cheek fullness
    c) Estrogen therapy does not change the fullness of cheeks
24) The most common change to lip fullness during feminizing hormone therapy is:
    a) Decreased lip fullness
    b) Increased lip fullness
    c) Estrogen therapy does not change lip fullness
25) Taking a higher dose of feminizing hormones than prescribed will:
    a) Increase how quickly changes happen to the skin
    b) Decrease how quickly changes happen to the skin
    c) Have no effect on how quickly changes happen to the skin
26) Most women experience a maximal effect of feminizing hormone therapy:
    a) Within 6 months of starting therapy
    b) After 1 year of starting therapy
    c) After 2 years of starting therapy
27) For which procedure(s) do you need maintenance therapy treatments:
    a) Laser hair reduction
    b) Electrolysis hair removal
    c) Both
    d) Neither

28) Laser hair removal techniques are able to reduce hair of what color(s):
    a) Dark-colored hair, such as black or brown
    b) Light-colored hair, such as blonde or gray
    c) All shades of hair
29) Electrolysis hair removal techniques are able to reduce hair of what color(s):
    a) Dark-colored hair, such as black or brown
    b) Light-colored hair, such as blonde or gray
    c) All shades of hair
30) Laser hair removal techniques can typically be used on which skin tone(s):
    a) Light shades of skin
    b) Dark shades of skin
    c) All shades of skin
31) Electrolysis hair removal techniques can typically be used on which skin tone(s):
    a) Light shades of skin
    b) Dark shades of skin
    c) All shades of skin
32) Which procedure(s) typically result in more permanent changes? (select all that apply)
    a) Facial neurotoxin injections, such Botox/Dysport/Xeomin
    b) Soft tissue facial fillers, such as Juvederm, Radiesse, or Sculptra
    c) Facial reconstruction
    d) Facial fat injections
33) Which procedure(s) typically result in more temporary changes? (select all that apply)
    a) Facial neurotoxin injections, such Botox/Dysport/Xeomin
    b) Soft tissue facial fillers, such as Juvederm, Radiesse, or Sculptra
    c) Facial reconstruction
    d) Facial fat injections
34) Which procedure(s) require that you be placed under general anesthesia, so that you are asleep during the procedure? (select all that apply)
    a) Facial neurotoxin injections, such Botox/Dysport/Xeomin
    b) Soft tissue facial fillers, such as Juvederm, Radiesse, or Sculptra
    c) Facial reconstruction
    d) Facial fat injections
